# Hereditary Syndromes Manifesting as Endometrial Carcinoma: How Can Pathological Features Aid Risk Assessment?

**DOI:** 10.1155/2015/219012

**Published:** 2015-06-16

**Authors:** Adele Wong, Joanne Ngeow

**Affiliations:** ^1^Department of Pathology and Laboratory Medicine, KK Women's and Children's Hospital, Singapore 229899; ^2^Cancer Genetics Service, National Cancer Centre Singapore, Singapore 169610; ^3^Oncology Academic Clinical Program, Duke-NUS Graduate Medical School, Singapore 169857; ^4^Division of Medical Oncology, National Cancer Centre Singapore, 11 Hospital Drive, Singapore 169610

## Abstract

Endometrial carcinoma is the most common gynecological tumor worldwide. It can be the presenting malignancy, acting as the harbinger, of an undiagnosed hereditary syndrome. Up to 50% of females with Lynch syndrome present in this manner. Differentiation between Lynch, Muir-Torre, and Cowden syndromes can at times be challenging due to the overlapping features. Our review emphasizes on the strengths, pitfalls, and limitations of microscopic features as well as immunohistochemical and polymerase chain reaction- (PCR-) based tests used by laboratories to screen for DNA mismatch repair (MMR) and *PTEN* gene mutations in patients to enable a more targeted and cost effective approach in the use of confirmatory gene mutational analysis tests. This is crucial towards initiating timely and appropriate surveillance measures for the patient and affected family members. We also review the evidence postulating on the possible inclusion of uterine serous carcinoma as part of the spectrum of malignancies seen in hereditary breast and ovarian carcinoma syndrome, driven by mutations in *BRCA1/2*.

## 1. Introduction

Many genetic mutations have been elucidated in the past half century leading to either the discovery or better understanding of hereditary syndromes associated with malignancies in the female genital tract. The discovery of the* BRCA1/2* gene in the early 1990s [1] and subsequent work on gene linkage analysis identified it as the main causative gene in hereditary breast and ovarian carcinoma (HBOC) syndrome [2]. Since then, other mutations in molecular pathways such as DNA mismatch repair (MMR) [3] and* PTEN* [4] have shown to result in syndromes causing endometrial carcinomas, the most common gynecological carcinoma to afflict women worldwide [5].

Gene mutations inherited in a Mendelian fashion have been associated with up to 10% of all malignancies occurring in humans [6]. As such, it is imperative that syndromes are identified in probands who present with malignancies to enable prompt initiation of appropriate counseling and testing for the individual and family to reduce morbidity and mortality amongst these individuals [7]. As some of these syndromes may have overlapping clinical features, clinicians or geneticists can be faced with a few possible differential diagnoses [8] as summarized in Table 1. In this aspect, close collaboration between oncologists, pathologists, and geneticists is necessary to ensure confirmatory genetic testing proceeds in a cost effective and timely manner for the patient and family members [9].

With the advent of immunohistochemical (IHC) markers and molecular testing for specific gene mutations, anatomic pathologists now play a bigger role than ever aiding oncologists and geneticists towards a more directed approach towards confirmatory genetic testing. This is particularly so for proband patients with sentinel tumors as the initial manifestation for any given family. Although risk assessment and predictive tools for various hereditary syndromes exist to aid clinician in identifying such patients, some patients fail to fulfill the criteria and are only picked up by pathologists during examination of the tumor tissue specimens.

In this review, we discuss these hereditary endometrial carcinoma syndromes and the important role gynecologists play in identifying at-risk patients as well as in the surveillance of such patients. We further place special emphasis on the role the pathologist plays in terms of appreciating the histological nuances present in tumor tissue using traditional light microscopy as well as the interpretation of newer ancillary investigations performed in the laboratory that may assist clinicians assessing potential patients with an underlying syndrome. We have included HBOC syndrome in this discussion as we wish to highlight the possible association of uterine serous carcinoma with this syndrome. The less common syndromes such as Muir-Torre syndrome and Cowden syndrome are emphasized as these may be missed if clinicians do not actively consider them when assessing patients.

## 2. DNA Mismatch Repair (MMR)

### 2.1. Lynch Syndrome

#### 2.1.1. Background

Lynch syndrome (LS), also known as hereditary nonpolyposis colorectal cancer (HPNCC), is an autosomal dominant syndrome [10, 11]. The incidence in the general population is estimated to be between 1 in 300 and 1 in 500 [12]. In LS, mutations in the DNA mismatch repair (MMR) gene result in widely dispersed replication errors or instability in highly repetitive error prone areas found primarily in intronic sequences of the genome, known as microsatellites [13]. Microsatellite instability (MSI) can be seen in patients harboring either germline or somatic DNA MMR gene mutations. LS is a result of germline mutations in the DNA MMR genes* MLH1, MSH2, MSH6, and PSM2* [14]. Nonhereditary somatic mutation is due to promoter hypermethylation of the* MLH1* gene resulting in silencing of the gene causing similar MSI levels in the genome seen in 10% to 25% of sporadic tumors, especially colorectal and endometrial carcinomas [15]. Unlike colorectal carcinomas, somatic mutations in the* BRAF* gene resulting in sporadic cases are far rarer in endometrial carcinomas [16, 17]. Germline deletions in a non DNA MMR gene,* EPCAM*, can result in inactivation of* MSH2* in approximately 1% of LS patients [18]. LS patients are at risk of developing colorectal cancer (80%), endometrial cancer (60%), ovarian cancer (12%), and other malignancies in the stomach, pancreas, upper urinary tract, biliary tract, and small intestines [19].

#### 2.1.2. Endometrial Carcinomas in Lynch Syndrome

Approximately 2% to 6% of all endometrial carcinomas can be attributed to germline mutations in the DNA MMR genes [20, 21]. Up to 50% of female patients with LS will present with endometrial carcinoma as their sentinel tumor [19, 22]. Germline mutation in the* MSH6* gene is associated with the highest risk for developing endometrial carcinomas [23, 24]. Mutations in* MLH1* and* MSH6* genes result in a higher risk of developing colorectal carcinoma [25]. Individuals with germline mutations in* PMS2* have the lowest overall risk of developing LS-associated tumors [26]. The median time for LS patients with endometrial carcinoma to develop a second tumor is estimated to be 11 years [27]. Therefore, identification of proband LS patients with endometrial carcinomas can result in timely and appropriate management to help reduce the potential of a second tumor in the patient or, in the case of her relatives, preventing tumors all together.

#### 2.1.3. Identification of Lynch Syndrome amongst Proband Patients with Endometrial Carcinoma

*(1) Clinical Evaluation*. LS patients have traditionally been identified by clinical assessment using validated criteria followed by confirmatory gene testing as described in Table 2. Sensitivity and specificity were increased in the 2004 revised Bethesda guidelines but still fell short due to the failure to specify gynecological tumors requiring further testing [28]. Among women with LS presenting with endometrial carcinomas, between 50% and 70% do not meet the Amsterdam or Bethesda guidelines due to the absence of a personal or family history suggestive of LS [20, 29–31]. Mutations in* MSH6* and* PSM2* are more likely to result in failure to meet either of the guidelines [29]. Clinical predictive tools relying on personal and family history such as PREMM_1,2,6_, MMRpredict, and MMRpro have been developed to quantify the risk of harboring germline DNA MMR mutations in colorectal patients [32]. Risk is determined by calculating the area under the receiver curve (AUC) with a ≥5% cutoff [33]. A large study involving 563 patients with endometrial carcinomas showed the three predictive tools to be having inferior sensitivity and specificity compared with IHC and polymerase chain reaction- (PCR-) based MSI analysis in identifying patients requiring confirmatory germline DNA MMR gene testing [33].

The deficiencies of the Amsterdam and Bethesda guidelines have resulted in the implementation of utilization of IHC and/or MSI analysis on tumor tissue to boost the ability to identify patients with LS. The Evaluation of Genomic Applications in Practice and Prevention (EGAPP) working group recommends IHC and MSA testing to be offered to all newly diagnosed colorectal carcinoma patients as part of the workup to identify all possible LS individuals [34, 35]. Currently, this proposal does not extend to include endometrial carcinoma patients. The Society of Gynecologic Oncologists (SGO) and National Comprehensive Cancer Network (NCCN) have only proposed blanket IHC and/or MSI analysis of women with endometrial carcinoma under the age of 50 years [36]. However, the suggestion to offer universal testing has been advocated as many proband LS patients present with endometrial carcinoma as their sentinel tumor without appropriate family history to trigger IHC and/or MSI analysis testing. Furthermore, the majority of LS patients will present with endometrial carcinoma at 50 years and above [20, 21, 37]. The SGO and NCCN guidelines very likely fail to optimally identify patients with LS and subsequent initiation of appropriate cancer surveillance.

*(2) Histological Evaluation.* Endometrial carcinomas associated with LS have been shown to exhibit a tendency to occur in the lower uterine segment (LUS) with up to third of such tumors attributed to this syndrome [38]. Histologically, LS-associated tumors have a diverse morphological appearance. The most common subtype is endometrioid carcinoma but serous carcinoma, clear cell carcinoma, and carcinosarcoma are also well accounted for [39]. Nonendometrioid carcinomas such as clear cell carcinoma, serous cell carcinoma, and carcinosarcoma are known to occur in LS patients at a younger age than is commonly seen in non-LS patients [40]. There is also a well-documented predisposition for LS-associated tumors to exhibit high grade features with a mixed histology which can at times represent a huge challenge to anatomic pathologists attempting to subtype the tumor components into the various neat categorical variants [41, 42]. Difficulty in separating the various tumor components comprising endometrioid, serous, and/or clear cell carcinomas is not uncommon in such situations [42]. Interestingly, tumors seen arising in the LUS have been shown to occasionally disclose histological and immunohistochemical features which are difficult to ascertain if the tumor is an endometrial or endocervical primary adenocarcinoma [38].

Among tumors with an endometrioid appearance, a few histological features have been shown to suggest the possibility of an underlying MSI. Undifferentiated and dedifferentiated endometrioid carcinomas have been associated with MSI, in particular* MLH1/PMS2* gene mutation, due to either promoter methylation or germline mutation [40, 46, 47]. Undifferentiated endometrioid carcinomas consist of solid sheets of round to polygonal cells with vesicular nuclei and prominent nucleoli without any evidence of gland formation [48]. A tumor is deemed to be dedifferentiated when areas of moderately or even well differentiated endometrioid carcinoma are discernible [48]. Another histological feature often associated with MSI, both in germline mutated or sporadic promoter methylated tumors, is a heavy lymphocytic infiltrate within and around the endometrial carcinomas [46, 49].

Synchronous ovarian and endometrial tumors have also been connected to MSI. The most common pattern is that of endometrioid carcinomas in both the endometrium and ovary [50–52]. However, some patients may exhibit synchronous clear cell or undifferentiated carcinomas [50]. Serous carcinomas are uncommon. Gynecologists practicing in centers conducting universal screening for LS using IHC should consider requesting for the test to be performed when encountering young patients with ovarian cell carcinomas. This is due to a strong association with LS in patients in this age group [50]. This should also be extended to patients with synchronous uterine endometrioid carcinoma and ovarian clear cell carcinoma as rare reports have been documented in MSI patients [47, 50].

*(3) Ancillary Laboratory Tests.* The current gold standard confirmatory test for LS is the expensive gene mutational analysis of DNA MMR genes [21]. Cost effective and readily available screening tools available in most laboratories are (1) IHC to look for abnormal loss of DNA MMR proteins, (2) MSI analysis by PCR to detect for increased microsatellite repeats in specific loci and when required, and (3)* MLH1* promoter methylation also utilizing PCR [21]. The usual initial workflow to identify DNA MMR genes is by IHC for the 4 DNA MMR proteins (*MLH1, PMS2, MSH2*,* and MSH6*) and/or MSI analysis [53]. Some centers perform both tests on every tumor specimen to maximize detection [20, 22, 54] as described in Table 3. Tumor testing by IHC and/or MSI analysis has been reported to generally detect abnormal DNA MMR protein expression in 15–25% of unselected patients with endometrial carcinomas [20, 33]. Subsequent to this, all MSI patients exhibiting loss of DNA MMR protein* MLH1* expression can be further segregated using* MLH1* gene methylation testing to identify those with as somatic promoter methylation and those most likely to benefit from confirmatory germline mutational analysis [53, 54]. In contrast to patients with colorectal cancers [16], patients with sporadic MSI* MLH1* methylated endometrial carcinomas do not benefit from additional testing for V600E mutation of the* BRAF* gene as less than 1% display this mutation [17, 55].

*(a) Immunohistochemistry (IHC).* IHC is performed on paraffin embedded tumor tissue. The sensitivity and specificity when using the four DNA MMR protein markers are 91% and 83%, respectively [56]. However, it is important to recognize that IHC will not detect germline mutations where the DNA MMR protein is produced but nonfunctioning as in the case of missense mutations [57].

Normal DNA MMR proteins function as heterodimer complexes by forming pairs of dimers with* MLH1* partnering* PMS2* and* MHS2* pairing up with* MSH6*, with* MLH1* and* MSH2* acting as obligatory partners [58, 59]. Mutation in one of the protein in the pairing results in concurrent loss of staining of its partner protein. Somatic mutation via epigenetic methylation silencing of the* MLH1* promoter gene results in loss of* PMS2* IHC staining. Epigenetic silencing can also occur in the* MSH2* gene following deletions in the* EPCAM* gene leading to loss of IHC staining in the partner protein,* MSH6* [18]. Germline mutations in* MSH6* or* PMS2* genes do not result in loss of IHC staining in its obligatory partner [57, 60]. Individual loss of* PMS2* or* MSH6* IHC staining indicates the possible germline mutations in the respective genes [57, 60]. As such, a two IHC panel utilizing* PMS2* and* MSH6* is feasible [61].

An abnormal IHC staining pattern is where there is a total loss of nuclear staining in tumor cells [10]. Normal staining is seen in lymphocytes, stroma, and normal endometrium and these act as internal positive controls. A common problem arising from normal staining of intratumoral lymphocytes is mistaking these for tumor cells, resulting in false positive result of normal retention of MSI in the tumor [62–64]. Another common challenge is the difficulty in assessing the heterogeneous staining nature of the* MSH6* IHC marker [63]. Only small areas may exhibit normal retention of nuclear staining. As such, tumor tissue of an adequate size should be selected for IHC staining. When accurate interpretation on a tissue block remains problematic despite repeated testing, an equivocal or inconclusive report will be rendered with the recommendation to consider an alternative test such as MSI analysis.

*(b) Polymerase Chain Reaction (PCR).* MSI analysis is a PCR test which measures errors in DNA replication caused by loss or abnormal function of the DNA MMR protein. It is performed on paraffin-embedded tumor tissue using either mononucleotides only or a combination of nucleotides and dinucleotides to amplify common sites of instability in the genome [35, 65, 66]. The Bethesda MSI panel consists of two mononucleotides and three dinucleotides (BAT25, BAT26, D2S123, D5S346, and D17S250) [67] and is still widely used despite two well-known pitfalls caused by the dinucleotides [66]. The National Cancer Institute (NCI) panel of five mononucleotide markers (BAT25, BAT26, NR21, NR24, and NR27) in contrast has been shown to be more effective and reproducible [62, 66, 68]. MS-stable (MSS) phenotype tumors will show normal microsatellite repeats as normal tissues [65]. MSI-high (MSI-H) tumors show microsatellite instability in two or more of the tested loci while MSI-low (MSI-L) tumors show instability at one locus [65]. Although MSI-H is seen when germline mutation occurs in any of the four DNA MMR genes, mutations in* MSH6* more frequently results in MSI-L or even MSS status [14, 20, 69, 70]. MSS* MSH6* mutated cases are more commonly seen with the use of the Bethesda panel [69, 70]. One study previously showed 11.8% (12/102) of MSI-H tumors retained normal IHC staining, of which 2/12 of the discordant cases were patients with endometrial carcinomas and a family history of LS [54]. Possible reduced detection rates can occur in centers only relying on IHC. As such, MSI analysis is concurrently used in conjunction with IHC for testing in some centers [54]. MSI analysis is not capable of differentiation between tumors with* MLH1* promoter methylation and germline mutation [65]. Tumors with loss of* MLH1* IHC staining will need to undergo an additional PCR-based test to detect for* MLH1* gene promoter methylation [15, 71]. A patient with a tumor which tests negative with the* MLH1* gene promoter methylation test should be encouraged to undergo germline mutation testing for LS [71].

*(c) Confirmatory Gene Mutational Analysis for Lynch Syndrome.* Direct gene sequencing using the traditional Sanger sequencing method to uncover mutations in DNA MMR genes is carried out in conjunction with multiplex ligation-dependent probe amplification (MLPA) [20, 21]. MLPA is utilized to detect large genomic rearrangements. MLPA is also used to detect deletions in the* EPCAM* gene, which results in somatic methylation of the* MSH2* gene [72].

Some gene mutational analysis results of the four DNA MMR genes will indicate missense mutations [73], the significance of which is often unknown and are classified under the “variants of uncertain significance” (VUS) category [74]. A certain proportion of patients with IHC and/or MSI analysis results suggestive of LS will have no mutations in* DNA MMR* genes [73]. Current sequencing protocols may not be sufficiently sensitive to identify such mutations which reside deep within the introns or promoter regions of the genes. Unknown novel epigenetic effects have also been postulated as a cause.

#### 2.1.4. Recommended Surveillance

One of the main aims of identifying proband patients with LS is to unearth unsuspecting relatives who are carriers of the deleterious DNA MMR genes. The general expert consensus is that surveillance for the patient and family members with LS is required [54, 75] as highlighted in Table 4.

We concentrate on endometrial surveillance required for the proband's female family members. Female relatives of LS proband patients who are suspected or confirmed to be DNA MMR mutation carriers should be offered the option to undergo surveillance in the effort to prevent endometrial carcinomas. There is some support for the use of transvaginal ultrasound and endometrial biopsy either annually or every 2 years from the age of 30 to 35 [76]. In a recent retrospective study over a 10-year period, gynecologists were the designated physicians to perform surveillance colonoscopy and endometrial curettage under sedation to reduce discomfort, at the same outpatient visit scheduled every 1 to 2 years apart [77]. Endometrial curettage usually provides a large amount of tissue compared to other biopsy methods and is, thus, an added advantage during histological examination. The 55 LS mutation carriers in this study had a combined 111 surveillance visits with 4.5% (5/111) of these visits resulting in abnormal biopsy findings. Four patients had complex hyperplasia and one patient was diagnosed with endometrioid carcinoma, FIGO grade 1 stage 1a. The patient with endometrioid carcinoma and three others with complex hyperplasia did not have thickened endometrium on transvaginal ultrasound to warrant a biopsy. With the findings by Nebgen et al. [77] in mind, it is prudent that if it is decided that no active surveillance is to be carried out, female carriers of the DNA MMR gene mutations must be educated on the need to seek immediate medical attention for further investigation if they have any abnormal uterine bleeding.

### 2.2. Muir-Torre Syndrome, a Variant of Lynch Syndrome

#### 2.2.1. Background

Muir-Torre syndrome (MTS) is now considered a subtype of LS [78, 79] with an estimated overall frequency of 9.2% among individuals with LS [80]. MTS is mostly due to germline mutations in* MSH2* and* MLH1* [81]. It is characterized by sebaceous gland neoplasms (except sebaceous hyperplasia) and keratoacanthoma with 57% of patients presenting with diagnostic skin lesions as their sentinel pathology [81, 82]. Recognition of MTS is a problem and may stem from patients considering these lesions as insignificant and not disclosing in their medical histories unless specifically asked [83]. It is important to be vigilant for new onset skin lesions in patients with a previous history of endometrial carcinoma. For patients who have never been tested for LS, these skin lesions may indicate the need to testing. In such instances, the patient's original endometrial tumor tissue blocks can be used for IHC or MSI analysis testing.

Endometrial carcinoma is not the most common visceral tumor to be associated with MTS. However, there have been early case reports in the literature clearly documenting endometrial carcinomas presenting as the sentinel tumor in a few patients with MTS [84, 85]. An old meta-analysis study uncovered 120 patients reported in the literature to have had internal malignancies, of whom seven patients were noted to have been diagnosed with endometrial carcinoma [86]. Colorectal carcinoma was shown to afflict almost half of all MTS patients with internal malignancies and a quarter was reported to have genitourinary tract malignancies [86].

In a similar vein to colorectal and endometrial carcinomas, IHC markers have been proposed as part of the work-up to identify patients presenting with sebaceous neoplasms requiring confirmatory DNA MMR gene mutational analysis [83, 87–89]. More recent studies have shown IHC to be less reliable when performed on sebaceous neoplasms that are on colon or endometrial carcinomas [89, 90]. IHC has been shown to have an unacceptably high false-positive rate of 52% with a positive predictive value (PPV) of 22% and negative predictive value (NPV) of 95% [90]. Variable results within individual patients with multiple sebaceous neoplasms have also been demonstrated [90]. Despite the pitfalls, some center may still opt to perform IHC on sebaceous neoplasms [89]. Individuals with germline* MSH6* mutations are associated with higher risk of developing endometrial carcinomas [23, 24] and generally do not conform to the classic LS presentation with personal and family histories of young-onset colorectal cancer [37].* MSH6* germline mutations are not uncommon in MTS patients [89, 90]. Selected IHC testing prompted by clinical history may potentially result in the missed the opportunity of identifying MTS patients harboring mutations in the* MSH6* gene.

## 3. PTEN

### 3.1. Cowden Syndrome

#### 3.1.1. Background

Cowden syndrome (CS) is an autosomal dominant syndrome with incomplete penetrance and variable expressivity characterized by multiple hamartomas, skin lesions, abnormal CNS lesions, and an increased risk of developing carcinomas in the breast, endometrium, thyroid, and genitourinary tract [4, 91]. CS is due to germline mutations in the phosphatase and tensin homologue (*PTEN*) gene located on chromosome 10q23.3 [92]. The incidence of CS in the general population is difficult to ascertain due to the variable and often subtle expression resulting in difficulty in diagnosing proband patients. A clinical epidemiological study utilizing confirmatory* PTEN* gene mutation analysis suggests a prevalence of between 1 in 200,000 and 1 in 250,000 [93] but is likely an underestimation [94].* PTEN* is a tumor suppressor gene containing 9 exons which encodes for a 403 amino acid protein [94]. The* PTEN* protein plays a role in the* PTEN* pathway by negatively regulating the PI3K/AKT/mTOR pathway by dephosphorylating phosphatidylinositol (3,4,5-)triphosphate (PIP3) to PIP2 [94]. This results in a decrease in downstream PI3K kinase activity such as phosphoinositide dependent kinase 1 (PDK-1), Akt, mTOR, and ribosomal protein s6 kinase (S6K1) [95–97]. The loss or reduction of* PTEN* activity results in activation by phosphorylation of important cellular proteins in key signaling pathways involved in cell cycle progression, metabolism, translation, growth, migration, invasion, angiogenesis, and apoptosis [95–97].

#### 3.1.2. Endometrial Carcinomas in Cowden Syndrome

Early tumorigenesis from benign endometrium to endometrial intraepithelial neoplasia has been shown to involve the loss of* PTEN* function [98, 99]. It is the most common gene mutation in endometrial carcinomas with approximately 40% reported to harbor* PTEN* gene somatic mutations [98, 99]. In two separate studies, between 16% and 17% of women with endometrial carcinomas who fulfill the clinical diagnostic criteria for CS have been shown to carry the deleterious germline* PTEN* mutations [100, 101].

Endometrial carcinoma is considered as one of the major criteria by NCCN for the diagnosis of CS. The lifetime risk of CS patients with* PTEN* germline mutation developing endometrial carcinomas is between 13% and 19%, which is a substantially higher lifetime risk of the general population estimated to be between 2% and 4% [102–105]. Among female individuals with CS, 12% will present to an oncology department with endometrial carcinoma as their sentinel malignancy [106]. Among female CS patients previously afflicted with a malignancy, 10% were shown represent at a later date with endometrial carcinoma as their second malignancy [106]. It must be stressing that, due to the inherent rarity of CS, the syndrome itself accounts for only a minor proportion of unselected endometrial carcinomas [107]. In a study of 240 unselected endometrial carcinoma patients, no germline mutation of* PTEN* was discovered [107]. In a recent study, CS and CS-like patients with endometrial carcinoma were shown to be linked to mutations other than the* PTEN* gene [108]. In women with CS, the majority of endometrial carcinomas have been noted to occur between the ages of 30 and 50 [101, 109]. However, there have been case reports of endometrial carcinomas occurring in CS patients under the age of 20, which is a highly unusual phenomenon in the general population [110–112]. These tumors have generally been endometrioid carcinomas [110–112] with one case preceded by the finding of an atypical polypoid adenomyoma in the uterine curetting [113].

The majority of reports indicate endometrioid carcinoma to be most common subtype to afflict women with CS [110–113]. However, in a large prospective multicenter study involving 371 CS and CS-like patients with endometrial carcinoma, only 42% were noted to have carcinoma of the endometrioid subtype [108]. The remaining 58% were reported to been diagnosed with a nonendometrioid carcinoma, of which 50% were labeled as endometrial carcinoma, NOS. Serous/clear cell carcinoma (5%), mucinous carcinoma (0.3%), and sarcoma (2.7%) were shown to account for the other malignancies in the nonendometrioid carcinoma group [108]. As all the patients were recruited based on an inclusion criterion of endometrial carcinoma, we assume that the sarcoma (2.7%) stated may in fact be carcinosarcomas [108]. Carcinosarcomas are recognized under the WHO tumor classification system to be of epithelial origin containing at least an area of malignant stromal transformation [48]. Unfortunately, the study was limited by the lack of central pathology review and we are therefore unable to use the reported histology subtypes to help guide clinical risk assessment for germline PTEN mutations. We hope that future studies will address this issue.

In addition to endometrial carcinoma, CS patients are also predisposed to uterine leiomyomata, benign ovarian cysts, and functional menstrual abnormalities [114, 115].

#### 3.1.3. Identification of Individuals with Cowden Syndrome

Clinical criteria remain the bedrock of identifying and diagnosing CS. The latest diagnostic criteria incorporating pathognomonic features with major and minor indications have been outlined by the 2014 NCCN [116]. A suggested workup for suspected individuals can be found in Table 3. Validated pediatric and adult risk calculators to determine the risk of an individual harboring* PTEN* gene mutations have been made freely available online (http://www.lerner.ccf.org/gmi/ccscore/) [101].

#### 3.1.4. Clinicopathological Features of Cowden Syndrome

*(1) Other Malignant Manifestations Seen in Individuals with CS.* The lifetime risk for breast carcinoma among patients with CS is between 25% and 50% [117]. Among women with CS presenting with breast carcinoma, 48% present with it as their first malignancy and of this group, and 22% are likely to represent with at a later date with a new primary breast carcinoma [106]. Histologically, there is a rare but highly distinctive and striking feature of CS-associated ductal adenocarcinomas seen on microscopy as dense hyalinized collagenous stroma. When present, this distinctive stroma can be seen enveloping the malignant cells [118]. The second most common malignancy among CS patients is thyroid carcinoma, with a reported lifetime risk of between 3 and 10% [119]. Thyroid carcinoma is the initial presenting tumor in 11% of CS patients [106] with an overrepresentation of follicular histology compared to the general populations [119]. Other malignancies such as renal cell carcinoma, melanoma, and colorectal and gastric carcinoma may occur [101, 103, 120, 121]. One study showed colorectal carcinoma to be present in 13% of CS patients, all of whom were <50 years at the time of diagnosis [120].

*(2) Benign Entities Seen in Individuals with CS.* The most characteristic benign lesion in CS is mucocutaneous hamartomas which can present at birth and, typically, by adulthood [115, 122–124]. Hamartomatous ganglioneuromatous, adenomatous, or hyperplastic polyps involving the gastrointestinal tract simply seen as polyps on endoscopy are another common finding affecting between 35% and 85% of CS patients [114, 120, 125–129]. Other benign lesions are gastritis and the stigmata of CS, esophageal glycogenic acanthosis [120, 128]. Benign thyroid lesions are also a common feature [119, 130, 131]. Meanwhile, CS patients often present with benign breast lesions ranging from fibrocystic breast disease to a characteristic mammary hamartoma-like lesion with densely hyalinised collagen [118, 132]. These lesions have a propensity to be multiple and bilateral [118]. Rarely, the lesion may be so extensive as to diffusely replace most of the normal breast tissue [118]. Identification of such lesion on histology should prompt the pathologist to inform the breast surgeon to clinically assess the patient for CS. This is especially pertinent if the patient presents with the mammary hamartomatous lesion and has yet to develop breast carcinoma. The pathognomonic lesion of CS is adult Lhermitte-Duclos disease, a slow growing hamartomatous outgrowth of the cerebellum [133, 134].

#### 3.1.5. Ancillary Laboratory Tests

*(1) Immunohistochemistry (IHC).* To date, only the* PTEN* IHC clone 6H2.1 by Dako has been shown to provide reproducible staining results with good kappa scores among pathologists assessing endometrial carcinomas to determine patients' eligibility for targeted chemotherapy [135–138]. Djordjevic et al. [137] showed* PTEN* protein IHC marker to be lost in 64% endometrial carcinomas, of which only 67% of these were subsequently proven to contain* PTEN* mutations. This study did not determine if the* PTEN* gene mutations identified were somatic or germline [138]. Hence, it can be inferred that* PTEN* protein IHC if used to identify patients requiring confirmatory gene mutational analysis for CS will result in a high level of false positives and, clinically, is not useful for the identification of patients at risk of CS.

*(2) Confirmatory Gene Mutational Analysis.* The majority of patients meeting the guidelines for the diagnosis of CS have a mutation of the* PTEN* gene (up to 80%) but the figure is lower when patients are referred from the community (approximately 25%) [101]. Germline mutations are seen throughout the 9 exons of the* PTEN* gene but most appear to cluster on exon 5 [139–144]. It has been shown that de novo* PTEN* mutations are found in approximately 40% of proband patients who fulfill the criteria of possibly harboring the* PTEN* gene mutation [145]. Mutations in genes such as* SDBH-D* and* KLLN* which can result in similar disruption to the* PTEN* gene are also seen in some CS patients [108, 119, 146]. These genetic and epigenetic factors can serve as phenotypic modifiers.

#### 3.1.6. Recommended Surveillance and Management

Current recommendations are highlighted in Table 5.

The 2014 NCCN guidelines currently indicate that women with CS aged 30–35 should be considered for annual random endometrial biopsies and/or ultrasound on an individual basis. These women must be educated on the need to respond to promptly symptoms [116]. The statement on the consideration of enrollment in a clinical trial to determine effectiveness and necessity of screening has been omitted [116]. The 2014 NCCN guidelines also suggest that risk-reducing hysterectomy is discussed as an option on a case-by-case basis [116]. However, in view of reports of endometrial carcinoma presenting even in adolescence with CS, any abnormal uterine bleeding should be an indication for an endometrial biopsy in all CS patients.

## 4. BRCA

### 4.1. Is Uterine Serous Carcinoma Part of the Spectrum of Hereditary Breast and Ovarian Carcinoma Syndrome?

#### 4.1.1. Background

Women with germline mutations in* BRCA1* and* BRCA2* genes have an increased lifetime risk of developing breast (40%–85%) and ovarian (10–39%) carcinomas [147]. Additionally, germline* BRCA1/2* mutations have also been associated with carcinomas of the fallopian tube, colon, melanoma, and pancreas [148–151].

*BRCA1* or* BRCA2* genes play a crucial role in maintaining the integrity of the genome via repair of DNA double stranded breaks using the homologous recombination pathways [152].* BRCA1* and* BRCA2* proteins link with* RAD51* at damaged DNA and recombination sites [153]. Cells with mutations in either* BRCA* gene result in hypersensitivity to crosslinkage agents such as cisplatin or poly (ADP-ribose) polymerase (PARP) inhibitors which produce double stranded breaks [154–156].

#### 4.1.2. Evidence for Inclusion of Uterine Serous Carcinoma as Part of the Spectrum of Hereditary Breast and Ovarian Carcinoma Syndrome

Uterine serous carcinoma (USC) is a high grade variant of endometrial carcinoma [157, 158]. It accounts for 10% of all endometrial carcinomas [159]. USC is an aggressive disease usually seen in older women with low estrogen states and is usually associated with widespread peritoneal involvement, advanced stage at initial presentation, and, prior to the introduction of platinum therapy, dismal survival rates [159, 160]. It shares similar prognosis and identical histological features with high grade serous carcinomas of the ovary and primary peritoneum [161]. Further evidence suggesting a possible underlying difference in biology special to USC was the finding of a higher incidence of subsequent breast cancer in women with USC compared to endometrioid endometrial carcinoma (25% versus 3.2%, resp., *P* < 0.001) [162].

Although USC is not currently recognized as a feature of any hereditary cancer syndrome, there have been previous speculations on its possible association with HBOC syndrome. It has previously been demonstrated that not only do serous carcinomas of the fallopian tube, uterus, and ovary resemble one another in histology and clinical behavior, comparative genomic hybridization has been shown to have strikingly similar mutations [163]. The endometrial carcinoma genomic characteristics published by the Tumor Cancer Genome Atlas (TCGA) also provide data to suggest a link between USC and serous carcinomas of the ovary [164]. Interestingly, recent evidence suggests most serous cell carcinoma of the ovarian carcinomas very likely arise from the epithelium fallopian tube which has undergone malignant transformation [165, 166]. Further support for this notion is the observation that USC responds to therapeutic agents used for ovarian and peritoneal serous carcinomas [160].

A case report in 1999 documented two Ashkenazi Jewish sisters, where one sister presented with postmenopausal USC followed by the other sister with ovarian serous carcinoma [167]. Both were later found to harbor one of the* BRCA1* founder mutations. This resulted in the postulation of a possible connection between USC and HBOC syndrome [167]. Following on to this, other studies have gone on to investigate this link with varying results [168–174] with all but one [174] concentrating either mainly or solely on sequencing for the founder mutations found in Ashkenazi Jews. A large Israeli study of 199 endometrial carcinoma Ashkenazi Jewish patients, mostly with endometrioid carcinoma, was negative for* BRCA1/2* mutations [169]. Seventeen patients in this study had been diagnosed with USC. Subsequent to this, four other studies involving only Jewish patients with USC found an increased mutation rate in* BRCA1* between 14% and 27% [170–173] which is significantly higher than the 2.3% mutation rate in Israeli population [175]. Between 50% and 100% of these patients with* BRCA1/2 *mutations either had a personal history of breast carcinoma or at least a first degree relative with breast carcinoma or ovarian carcinoma [170–173].

However, a Canadian study involving 56 non-Jewish women with USC failed to detect any of the four commonest* BRCA1* or* BRCA2* mutations, of which three are founder mutations in Ashkenazi Jews [168]. A recent study involving 151 non-Jewish patients with USC found the frequency of* BRCA1* germline mutations to be 2% compared to the general non-Ashkenazi American population of 0.06% [174, 176]. A comprehensive search for all classes of* BRCA1/2* mutations was performed using the BROCA panel. Interestingly, 36 other patients had nondeleterious or variants of unknown significant mutations, 12 of which are reported as benign on the Breast Cancer Information Core database (BIC). It would be important that as longitudinal studies of patients with BRCA mutations mature it will help clarify if USC is truly a* BRCA*-related tumor.

#### 4.1.3. Recommended Surveillance

Current surveillance guidelines are described in Table 6.

Risk reducing surgeries have led to cancer specific survival benefit as well as a reduction in all causes of mortality [177] but remain unclear if patients electing for risk reducing salpingo-oophorectomy should have concurrent opportunistic hysterectomy. However, in view of the rarity of USC as a whole, the absolute risk of developing it is likely to be small. It does not seem warranted at this point to recommend for women to undergo hysterectomy as part of primary prevention of BRCA-associated USC. However, other more crucial issues as indicated by the recent findings in the GOG-0199 study suggest postmenopausal age, abnormal transvaginal ovarian ultrasound findings and elevated serum CA-125 are associated with elevated risk of harboring invasive serous carcinoma in fallopian tube, ovary, and peritoneum or serous tubal intraepithelial carcinoma (STIC) in the fallopian tube of asymptomatic patients with deleterious* BRCA1/2* gene mutations [177]. Not to be taken lightly is also the host of psychological issues faced by such patients [178]. These need to be taken into account when arriving at a clinical decision if such women should be advised to have a hysterectomy and possibly bilateral salpingo-oophorectomy.

## 5. Conclusion

Endometrial carcinomas can be the first presentation of an underlying hereditary cancer syndrome. Endometrial carcinoma can arise in patients with LS and in lesser known conditions such as MTS, CS, and possibly HBOC. Clinicians and pathologists alike play vital roles in identifying who may require genetic testing by better understanding the associated malignant and nonmalignant features of these conditions and the pitfalls of existing diagnostic tests. To better understand how best to screen these high-risk patients for endometrial carcinomas, we will need further research.

## Figures and Tables

**Table 1:** A summary of the epidemiological, mutational, clinical, and pathological characteristics and features encountered in hereditary syndromes manifesting as endometrial carcinoma. The indicators noted by pathologists to augur a need to notify clinicians on the possible need for referral to a geneticist for further clinical assessment and confirmatory gene mutational testing. Highlighted in the extreme right column are the histological features seen on microscopy and ancillary tests including immunohistochemistry and polymerase chain reaction- (PCR-) based tests such as microsatellite instability analysis and MLH-1 methylation study.

Syndrome	Incidence in general population	Lifetime risk of developing endometrial carcinoma	Most common sentinel tumor in women (%)	Germline gene mutation	Associated malignancies	Indicators to prompt pathologist to alert clinician on the possible need for referral to a geneticist
Lynch syndrome or hereditary nonpolyposis syndrome (HNPCC)	1 in 300 to 1 in 500	40%–60%	Endometrial carcinoma (50%)	*MLH1*, *MSH2*, *MSH6*, *PSM2*, *EPCAM *	Endometrial, ovarian, gastric, breast, intestinal, pancreatic, urinary tract, renal, and bile duct carcinoma	**Microscopic findings**: Mixed carcinoma. Undifferentiated or differentiated carcinomas. Significant peritumoral and/or intratumoral lymphocytic infiltrate. Tumors arising from lower uterine segment. Synchronous endometrial and ovarian carcinomas. **Ancillary investigations**: Immunohistochemistry: Loss of MMR protein staining for MLH1, MSH2, MSH6 or PSM2. Methylation study: Absence of promoter methylation of MLH1 in cases with loss of IHC staining. Microsatellite instability analysis: Instability of >1 (MSI-H) or instability in 1 locus (MSI-L)

Muir-Torre syndrome	Variant of Lynch syndrome with an incidence of 9% among Lynch syndrome patients	20%–60%	Colorectal carcinoma (47%) #if benign lesions also taken into account, the most common sentinel tumor is sebaceous neoplasms (50%)	Similar to Lynch syndrome	Similar to Lynch syndrome	**Microscopic findings**: Sebaceous neoplasm: sebaceous adenoma, sebaceoma, sebaceous carcinoma. (sebaceous hyperplasia not shown to be associated with Muir-Torre syndrome). **Ancillary investigations**: (1) Immunohistochemistry: loss of MIHC staining for MLH1, MSH2, MSH6 or PSM2. (Poor positive predictive value without proper clinical assessment for syndrome).

Cowden syndrome	Estimated at 1 in 200,000 (likely underestimated due to difficulty in identifying such patients)	~28%	Breast carcinoma (48%) #if benign lesions are also taken into account, the most common sentinel tumor are mucocuteneous lesions (~85%)	*PTEN *	Breast, thyroid, and endometrial carcinoma	**Microscopic findings**: Trichilemmommas, particularly if multiple Breast hamartoma, especially if prominent hyalinised stroma. Breast carcinoma with dense hyalinised collagenous stroma. Multiple hamartomatous gastrointestinal polyp. Oesophageal glycogenic acanthosis. Histologic confirmation of radiological suspicion of Lhermitte-Duclos. **Ancillary investigation**: Immunohistochemistry: PTEN IHC loss in trichilemmommas has not been shown to be particularly helpful in identifying cases as it is also commonly lost in sporadic cases as well.

MSI-L, microsatellite low; MSI, microsatellite high; and MMR, mismatch repair genes (i.e., *MLH1*, *MSH2*, *MSH6*, and *PMS2*).

**Table 2:** Amsterdam I and II criteria as well as the Revised Bethesda Guidelines for diagnosis of Lynch syndrome. The Revised Bethesda Guidelines was developed with the intention of identifying individuals who should undergo investigation for Lynch syndrome by evaluation of MSI analysis and/or immunohistochemistry (IHC) testing of their tumors. (Adapted from [43]).

**Amsterdam I criteria **	
(i) Three or more relatives with histologically verified colorectal cancer, one of which is a first-degree relative of the other two. Familial adenomatous polyposis should be excluded.	
(ii) Two or more generations with colorectal cancer.	
(iii) One or more colorectal cancer cases diagnosed before the age of 50 years.	

**Amsterdam II criteria **	
(i) Three or more relatives with histologically verified Lynch syndrome-associated cancer (colorectal cancer, cancer of the endometrium, small bowel, ureter, or renal pelvis), one of which is a first-degree relative of the other two. Familial adenomatous polyposis should be excluded.	
(ii) Cancer involving at least two generations.	
(iii) One or more cancer cases diagnosed before the age of 50 years.	

**Revised Bethesda Guidelines **	
(i) Colorectal carcinoma diagnosed at younger than 50 years.	
(ii) Presence of synchronous or metachronous colorectal carcinoma or other Lynch Syndrome-associated tumors^#^.	
(iii) Colorectal carcinoma with MSI-high pathologic-associated features (Crohn-like lymphocytic reaction, mucinous/signet cell differentiation, or medullary growth pattern) diagnosed in an individual younger than 60 years old.	
(iv) Patient with colorectal carcinoma and colorectal carcinoma or Lynch syndrome-associated tumor diagnosed in at least 1 first-degree relative younger than 50 years old.	
(v) Patient with colorectal carcinoma and colorectal carcinoma or Lynch syndrome-associated tumor^#^ at any age in two first-degree or second-degree relatives.	

^#^Lynch syndrome-associated tumors include tumor of the colorectum, endometrium, stomach, ovary, pancreas, ureter, renal pelvis, biliary tract, brain, and small bowel.

Caveat: Muir Torre syndrome is considered a subset of Lynch syndrome with patients also having sebaceous neoplasms and/or keratoacanthomas.

**Table 3:** Endometrial carcinoma testing result using MSI analysis and/or immunohistochemistry with additional testing strategies for Lynch Syndrome. Additional suggested testing strategies for patients who have been tested using either MSI analysis and/or immunohistochemistry with a four-panel marker (*MHL1, MSH2, MSH6, and PMS2*) or a ^#^two-panel marker (MSH6 and PMS2) (adapted from [43]).

MSI analysis	Immunohistochemistry protein expression				Possible causes	Further action
	MLH1	MSH2	MSH6^#^	PMS2^#^		
MSS/MSI-L	+	+	+	+	Sporadic carcinoma	None.
MSI-H	+	+	+	+	Germline mutation in MMR or EPCAM genes	MLH1, MSH2, then MSH6, PMS2, and EPCAM genetic testing
MSI-H	NA	NA	NA	NA	Sporadic or germline mutation in the MMR or EPCAM genes	Consider IHC to guide germline testing if IHC is not done germline testing of MLH1, MSH2, MSH6, PMS2, and EPCAM genes
MSI-H or NA	−	+	+	−	Sporadic cancer or germline mutation of MLH1	MLH1 promoter methylation testing. MLH1 genetic testing if absent hypermethylation or if testing not done
MSI-H or NA	−	+	+	+	Germline mutation MLH1	MLH1 genetic testing
MSI-H or NA	+	+	+	−	Germline mutation of PMS2, rarely MLH1	PMS2 genetic testing if negative MLH1 testing
MSI-H or NA	+	−	−	+	Germline mutation of MSH2 or EPCAM, rarely of MSH6	MSH2 genetic testing, if negative EPCAM, if negative MSH6
MSI-H or NA	+	−	+	+	Germline mutation of MSH2	MSH2 genetic testing if negative EPCAM testing
MSI-H, MSI-L or MSS	+	+	−	+	Germline mutation of MSH6, less likely MSH2	MSH2 genetic testing if negative MSH6 testing

MSI-L, microsatellite low; MSI, microsatellite high; MMR, mismatch repair genes (i.e., *MLH1*, *MSH2*, *MSH6*, and *PMS2*); NA, not available; +, protein expression present in tissue; and −, protein expression not present in tissue.

**Table 4:** Guidelines for screening at-risk or affected persons with Lynch syndrome. Recommendations are based on the strength of confidence and Grades of Recommendation, Assessment, Development, and Evaluation (GRADE). GRADE is a well-accepted rating of evidence relying on expert consensus about whether new research is likely to change the confidence level (CL) of recommendations (adapted from [43]).

Intervention	Recommendation	Strength of recommendation
Colonoscopy	Every 1 to 2 years beginning at age 20 to 25 or 2 to 5 years younger than youngest age at diagnosis of colorectal carcinoma in family if diagnosis before age 25. Considerations: start at age 30 in MSH6 and age 35 in PMS2 families Annual colonoscopy in MMR mutation carriers	Strong recommendation: Level of evidence (III): well-designed and conducted cohort or case-controlled studies from more than 1 group with cancer #GRADE rating: moderate

Pelvic examination with endometrial sampling	Annually beginning at age 30 to 35	Offer to patient: Level of evidence (V): expert consensus #GRADE rating: low

Transvaginal ultrasound	Annually beginning at age 30 to 35	Offer to patient: Level of evidence (V): expert consensus #GRADE rating: low

Esophagogastroduodenoscopy with biopsy of the gastric antrum	Beginning at age 30 to 35 and subsequent surveillance every 2 to 3 years can be considered based on patient risk factors	Offer to patient: Level of evidence (V): expert consensus #GRADE rating: low

Urinalysis	Annually beginning at age 30 to 35	Consideration: Level of evidence (V): expert consensus #GRADE rating: low

**Table 5:** Recommendations for diagnostic workup and cancer surveillance in patients with PTEN mutations. (Adapted from [44]).

	Paediatric (<18 years)	Adult female	Adult male
Baseline workup	(i) Targeted history and physical examination (ii) Baseline thyroid ultrasound (iii) Dermatologic examination (iv) Formal neurologic and psychological testing	(i) Targeted history and physical examination (ii) Baseline thyroid ultrasound (iii) Dermatologic examination	(i) Targeted history and physical examination (ii) Baseline thyroid ultrasound (iii) Dermatologic examination
Cancer surveillance			
From diagnosis	(i) Annual thyroid ultrasound (ii) Skin examination	(i) Annual thyroid ultrasound (ii) Skin examination	(i) Annual thyroid ultrasound (ii) Skin examination
From age 30^*∗*^	As per adult recommendations	(i) Annual mammogram (for consideration of breast MRI instead of mammography if dense breasts) (ii) Annual endometrial sampling or transvaginal ultrasound (or from 5 years before age of earliest endometrial cancer)	
From age 40^*∗*^	As per adult recommendations	(i) Biannual colonoscopy^**^ (ii) Biannual renal ultrasound/MRI	(i) Biannual colonoscopy^**^ (ii) Biannual renal ultrasound/MRI
Prophylactic surgery	Nil.		

^*∗*^Surveillance may begin 5 years before the earliest onset of a specific cancer in the family but not later than the recommended age cutoff.

^*∗∗*^The presence of multiple nonmalignant polyps in patients with PTEN mutations may complicate noninvasive methods for colon evaluation. More frequent colonoscopy should be considered for patients with a heavy polyp burden.

**Table 6:** Recommended surveillance and management of individuals with hereditary breast and ovarian carcinoma syndrome family members (from [45]).

Women	
(i) Breast awareness (periodic and consistent breast self-exam) starting at age 18.	
(ii) Clinical breast exam, every 6 to 12 months, starting at age 25.	
(iii) Breast screening	
(a) Age 25–29, annual MRI screening (preferred) or mammogram if MRI is unavailable based on earliest age of onset in family.	
(b) Age >30 to 75, annual mammogram and breast MRI screening.	
(c) Age >75, management should be considered on an individual basis.	
(iv) Discuss the option of risk reducing mastectomy	
(a) Counseling may include a discussion regarding degree of protection, reconstruction options, and risk.	
(v) Recommend risk-reducing salpingo-oophorectomy, ideally between 35 and 40 years of age and upon completion of child bearing or individualized based on earliest age of onset of ovarian carcinoma in the family.	
(a) Counseling includes a discussion of reproductive desires, extent of cancer risk, degree of protection for breast and ovarian cancer, management of menopausal symptoms, possible short term hormone replacement therapy (HRT) to recommend maximum age of natural menopause, and related medical issues.	
(vi) Address psychological, social, and quality-of-life aspects of undergoing risk-reducing mastectomy and/or salpingo-oophorectomy.	
(vii) For those patients who have not elected risk-reducing salpingo-oophorectomy, consider transvaginal ultrasound (preferably day 1 to day 10 of menstrual cycle in premenopausal women) and CA-125 (preferably after day 5 of menstrual cycle in premenopausal women), every 6 months starting at age 30 or 5 to 10 years before earliest age of first diagnosis of ovarian cancer in the family.	
(viii) Consider chemoprevention options for breast and ovarian cancer, including risks and benefits.	
(ix) Consider investigational imaging and screening studies, when available (e.g., novel imaging technologies and more frequent screening intervals) in the context of a clinical trial.	

Men	
(i) Breast self-exam training and education starting at age 35.	
(ii) Clinical breast exam every 6 to 12 months, starting at age 25.	
(iii) Consider baseline mammogram at age 40; annual mammogram if gynaecomastia or parenchyma/glandular breast density on baseline study.	
(iv) Starting at age 40:	
(a) Recommend prostate cancer screening for BRCA2 carriers.	
(b) Consider prostate cancer screening for BRCA1 carriers.	

Men and women	
(i) Education regarding signs and symptoms of cancer(s), especially those associated with BRCA gene mutations.	

Risk to relatives	
(i) Advise about possible inherited cancer risk to relatives, options for risk assessment, and management.	
(ii) Recommend genetic counseling and consideration of genetic testing for at-risk relatives.	

Reproductive options	
(i) For couples expressing the desire that their offspring not carry a familial BRCA mutation, advise about options for prenatal diagnosis and assisted reproduction, including preimplantation genetic diagnosis. Discussion should include known risks, limitations, and benefits of these technologies.	
(ii) For BRCA2 mutation carriers, there is a risk of a rare (recessive) Fanconi anaemia/brain tumor phenotypes in offspring if both partners carry a BRCA2 mutation should be discussed.	
